# Molecular Docking Studies and In Vitro Activity of *Paliurus spina-christi* Mill Extracts as Pancreatic Lipase Inhibitors

**DOI:** 10.3390/antiox13020160

**Published:** 2024-01-26

**Authors:** Fedora Grande, Mariangela Marrelli, Valentina Amodeo, Maria Antonietta Occhiuzzi, Iulia Pinzaru, Mary Fucile, Cristina Adriana Dehelean, Ersilia Alexa, Filomena Conforti, Giancarlo Statti

**Affiliations:** 1 Department of Pharmacy, Health and Nutritional Sciences, University of Calabria, 87036 Rende, Cosenza, Italy; fedora.grande@unical.it (F.G.); mariangela.marrelli@unical.it (M.M.); valentina.amodeo@unical.it (V.A.); mariaantonietta.occhiuzzi@unical.it (M.A.O.); fucilemary.fm@gmail.com (M.F.); giancarlo.statti@unical.it (G.S.); 2 Faculty of Pharmacy, Victor Babes University of Medicine and Pharmacy, 2nd Eftimie Murgu Sq., 300041 Timisoara, Romania; iuliapinzaru@umft.ro (I.P.); cadehelean@umft.ro (C.A.D.); 3 Faculty of Food Engineering, University of Life Sciences King Mihai I of Romania, Calea Aradului 119, 300641 Timisoara, Romania; ersiliaalexa@usab-tm.ro

**Keywords:** flavonoids, obesity, phytocomplexes, Rhamnaceae

## Abstract

Obesity is a risk factor for the onset of chronic diseases. One of the most promising approaches to treating obesity consists of reducing dietary fat absorption using extracts from plants because they contain phenolic compounds, especially flavonoids. *Paliurus spina-christi*, belonging to the Rhamnaceae family, is one of the five species belonging to the *Paliurus* genus. Herein, the aerial parts of the plant were extracted with methanol through the pressurized cyclic solid-liquid extraction using the Naviglio extractor^®^. The extracts were analyzed with High Performance Thin Layer Chromatography and investigated for their in vitro biological potential. The phytochemical analysis revealed that rutin has been shown to be the most abundant flavonoid component. The best antiradical activity was observed for the fruit extract with an IC_50_ value of 53.41 ± 1.24 µg/mL. This extract also has a better inhibitory capacity on lipid peroxidation evaluated at a different time of incubation. Potent lipase inhibitor activity of the extract from fruits was also demonstrated with in vitro experiments. This property can be attributed to a direct interaction of main components of *P. spina-christi* extract with the human pancreatic enzyme as demonstrated by the results of molecular docking experiments conducted on the crystallographic structures of lipase.

## 1. Introduction

Obesity is one of the toughest global public health challenges of the twenty-first century. In adulthood, this metabolic disorder represents an important risk factor for several diseases associated with premature death worldwide, including cardiovascular diseases, several types of cancer, diabetes, and osteoarthritis. Moreover, it has been demonstrated that excessive body fat may enhance oxidative stress [1]. As a consequence, generated free radicals may be involved in the etiology and development of obesity-related co-morbidities. Recent evidence indicates that antioxidant nutrients may have a protective effect [2]. Preventing obesity directly benefits health and well-being, both in childhood and in adulthood. Obesity is associated with an imbalance in energy metabolism, which leads to excessive fat accumulation in adipose tissue. Therefore, one of the most promising approaches to treating obesity consists of reducing dietary fat absorption. In this regard, the inhibition of pancreatic lipase, an enzyme responsible for the hydrolysis of dietary fats, has proven effective [3]. Since pancreatic lipase is involved in the conversion of 50–70% of triglycerides into monoglycerol and free fatty acids, inhibitors of this enzyme prevent triglycerides digestion and reduce fat absorption. The undigested fats will then be excreted in the stool. The mechanism of action of orlistat, a clinically used anti-obesity agent, is based on this principle: the drug binds to the catalytic site of pancreatic lipase, inhibiting its enzymatic activity [4]. However, this drug exerts several gastrointestinal side effects including abdominal pain, fatty or oily stool, diarrhea and increased defecation. As a result, the development of novel and effective therapeutic agents for the treatment of obesity with less or no adverse effects is still demanding.

Plants are still valuable sources of substances that can be used to prevent or treat diseases. In fact, it is known that plants contain nutritional compounds, including essential vitamins, and powerful bioactive molecules with proven therapeutic efficacy against many disorders. To date, 50% of accepted medicines derive from plants.

*Paliurus spina-christi* Mill. belongs to the Rhamnaceae family [5]. The name “Paliurus” derives from the Greek πᾰλιν (pálin) meaning again, and from οὖρον (oúron) literally orina, thus indicating “which makes one urinate again”, therefore “diuretic”. On the other hand, “spina-christi” comes from the belief that the crown of thorns placed on Christ’s head during the crucifixion was made of branches of this shrub [4]. This species is distributed in Southern Europe, Turkey, Iran, Iraq, and Syria [6]. In Turkey, the decoction of the seed of this plant were used a traditional remedy for nephralgia, kidney stones, diabetes, and as a diuretic [7]. The fruits of this plant for their diuretic properties, are used in phytotherapy for the preparation of infusions, which contribute to the elimination of uric acid, and for the preparation of cosmetic formulations for the treatment of oily skin. The fruit is edible and has a sour taste, reminiscent of a dried apple. Roasted and ground fruits were used as a coffee substitute. It is also a melliferous plant [8].

A number of studies have demonstrated several biological properties of this plant including antimicrobial, antioxidant, anti-inflammatory and enzyme inhibition [9,10,11,12], antidiabetic [13] and cytotoxic against breast cancer activity [14]. 

The antidiabetic activity of the plant was extensively investigated both in vitro, through the assessment of the e α-glucosidase and α-amylase inhibitory potential of the fruit extracts and some pure isolated components [6], and in vivo, on diabetic rats induced by streptozotocin [13]. The fruits extract was also demonstrated to improve the glucose uptake and to activate the insulin signaling pathways in HepG2 insulin-resistant cells [15]. Previous studies conducted to determine the phytochemical profile of this plant have demonstrated the presence of flavonoids in all its parts [16].

In this context, given the enzyme inhibitory properties already reported for this plant, particularly against α-glucosidase and α-amylase enzymes [6], in the present study, the aerial parts (leaves and fruits) of *P. spina-christi* were investigated for their inhibitory activity against pancreatic lipase. The antioxidant potential was verified as well. 

Several epidemiologic studies demonstrated the close relationship between obesity and diabetes. The term ‘diabesity’ refers to the link between these two metabolic disorders, as they are both characterized by defects of insulin action. Several metabolic defects are common to both obesity and diabetes, such as androgen dysfunction, sleep disturbances, impaired tissue perfusion and altered Vitamin D levels [17].

The investigated biological activities have been correlated to the presence of specific flavonoids, finding support in the results of molecular docking studies carried out in order to evaluate the capability of the phytocompounds to interact with the three-dimensional structure of the enzyme.

## 2. Materials and Methods

### 2.1. Chemicals

Lipase type II from porcine pancreas, Orlistat, 4-nitrophenyl caprilate (NPC), DPPH, ascorbic acid, β-carotene, linoleic acid, Tween 20, propyl gallate, and reference compounds utilized in HPTLC analyses were purchased from Sigma-Aldrich S.p.A. (Milan, Italy). Normal phase glass plates were obtained from Merck (Darmstadt, Germany). The utilized solvents were reagent grade and were purchased from VWR International s.r.l. (Milan, Italy).

### 2.2. Preparation of P. spina-christi Extracts

*P. spina-christi* aerial parts (fruits and leaves) were collected in Southern Italy, Calabria region in July 2021. A voucher specimen (leg. det. Carmine Lupia) was deposited at the Mediterranean Etnobotanical Conservatory, Sersale, Catanzaro (position number 15 of the Rhamnaceae section). Plant material (100 g each) was air-dried and extracted with methanol through Naviglio extractor^®^ (Atlas Filtri SrL, Limena, PD, Italy), using a plant:solvent ratio 1:10 g/mL × 2 cycles. Both obtained total extracts were dried under reduced pressure at 40 °C, weighed, and stored at 4 °C until analyses. 

### 2.3. High Performance Thin Layer Chromatography (HPTLC) Analysis

*P. spina-christi* fruits and leaves extracts were analyzed by High Performance Thin Layer Chromatography (HPTLC). The utilized CAMAG apparatus (Muttenz, Switzerland) consisted of a Linomat 5 sample applicator connected to a TLC Visualizer. Normal phase glass plates 20 cm × 10 cm were utilized (silica 2–10 µm; 2 µm thickness). Operating conditions were the same as previously described [18]. Plates were developed using a mixture ethyl acetate/dichloromethane/acetic acid/formic acid/water (100:25:10:10:11, *v*/*v*/*v*/*v*/*v*). For post-chromatographic derivatization, plates were dipped in freshly prepared NPR reagent (1 g diphenylborinic acid aminoethylester in 200 mL of ethylacetate) and anisaldehyde reagent (1.5 mL p-anisaldehyde, 2.5 mL H_2_SO_4_, 1 mL AcOH in 37 mL EtOH). Plates were then examined under a UV light at 254 or 366 nm and under white light upper and lower (WRT) before and after derivatization.

### 2.4. 2,2-Diphenyl-1-picrylhidrazyl (DPPH) Photometric Assay

The free radical scavenging activity of *P. spina-christi* fruit and leaf extracts was evaluated using the well-established DPPH test [18]. Briefly, 1600 µL of a 0.1 mM methanolic solution of the radical DPPH were added with 400 µL of different concentrations of the samples ranging from 5 to 1000 µg/mL. Ascorbic acid was used in the positive control group and absorbance was measured at 517 nm after 30 min of incubation in the dark. The IC_50_ values were determined by plotting the sample concentrations against percent values.

### 2.5. β-Carotene-Linoleate Bleaching Model System

This assay was carried out as earlier described [18]. In brief, β-carotene (5 mg) was dissolved in 25 mL of chloroform (0.2 mg/mL) and one milliliter of the solution was added to 0.02 mL of linoleic acid and 0.2 mL of 100% Tween 20. Chloroform was removed and distilled water (100 mL) was added. Five mL of the resulting emulsion were transferred into tubes containing 0.2 mL of test samples (concentration range 0.25–100 µg/mL) and incubated in a water bath at 45 °C. Oxidation of the emulsion was monitored spectrometrically by measuring the absorbance at 470 nm at the initial time and after 30- and 60-min. Propyl gallate was used in the positive control group. The antioxidant activity was calculated in terms of the successful prevention of β-carotene bleaching. The sample concentrations were plotted against percent values, and IC_50_ values were determined.

### 2.6. Pancreatic Lipase Inhibitory Activity

Lipase inhibitory activity was evaluated according to the method described previously [19]. *P. spina-christi* fruit and leaf extracts (25 µL) at concentration ranged from 10 to 0.0625 mg/mL were added to 5 mM 4-nitrophenyl caprilate (NPC, 25 μL), Tris–HCl buffer (pH = 8.5, 1 mL) and type II crude porcine pancreatic lipase (25 μL) 1 mg/mL solution in distilled H_2_O. The mixture was incubated at 37 °C for 25 min and then, the absorbance was measured at 412 nm with Perkin-Elmer Lambda 40 UV/VIS spectrophotometer (VWR International, Milan, Italy). The inhibitory activity (I) was calculated according to the following formula:

Inhibitory activity (I%) = 100 − [(B − b)/(A − a) × 100]

where A is the activity without inhibitor; a is the negative control without inhibitor; B is the activity with inhibitor; and b is the negative control with inhibitor. DMSO was used as negative control, and its activity was also examined.

The inhibitory concentration of the samples required to inhibit the activity of the enzyme by 50%, IC_50_, was calculated by regression analysis and Orlistat at 18 µg/mL was utilized as positive control.

### 2.7. Molecular Docking

Molecular docking was performed on the crystallographic structure of the human pancreatic lipase (HPL) corresponding to PDB entry 1LPB, in which the protein is in complex with two molecules of MUP, a C11 alkyl phosphonate inhibitor [20]. The molecular structures of rutin, quercitrin, kaempferol-3-*O*-glucoside-7-*O*-rhamnoside, quercetin and kaempferol were built by using the modeling software Avogadro 1.2.0 [21]. Docking calculations were performed by using AutoDock Vina 1.1.2 [22]. Preliminary conversion of the structures from the PDB format was carried out by the graphical interface AutoDock Tools 1.5.6 [23]. During the conversion, polar hydrogens were added to the crystallographic enzyme structures, whereas apolar hydrogens of the ligands were merged with the carbon atom they were attached to. Full flexibility was guaranteed for the ligands, resulting in sixteen for rutin, ten for quercitrin, fifteen for kaempferol 3-*O*-glucoside-7-*O*-rhamnoside, six for quercetin and five active torsions for kaempferol. For all the compounds, each simulation was carried out at very high exhaustiveness. The binding modes of the ligands were analyzed through visual inspection, and intermolecular interactions were evaluated by using the automated protein-ligand interaction profiler, PLIP [24] and VMD (Visual Molecular Dynamics) [25].

### 2.8. Statistical Analysis

All the experiments were performed in triplicate and data were expressed as mean ± S.E.M. The normality of data and homogeneity of variances were checked using D’Agostino-Pearson’s K2 test and Levene’s test. Non-linear regression analyses were performed with Graph-Pad Prism 5 (Graph Pad Software Inc., San Diego, CA, USA). Statistical differences were tested by one-way analysis of variance (ANOVA). Differences were considered significant for *p* < 0.05 (Sigma Stat Software 3.5, Systat Software Inc., San José, San Rafael, CA, USA). Pairwise post-hoc comparisons were performed using the Bonferroni post-hoc test, while the Dunnett’s multiple comparison test was used to compare treated and control groups (SigmaPlot 12.0, Systat Software Inc., San José, CA, USA).

## 3. Results and Discussion

### 3.1. Phytochemical Constituents

The aerial parts (fruits and leaves) from *P. spina-christi* were collected in Calabria (Southern Italy) and extracted with methanol through Naviglio extractor^®^.

This technique is based on a new principle of rapid solid-liquid extraction (Naviglio’s principle), which, when compared to traditional applications, allows a significant reduction of extraction times, generally leads to higher yields, does not require system heating, facilitates the extraction of active ingredients, and avoids their degradation.

A higher yield (42.3%) was obtained from the leaves compared to that from the fruits (3.7%). The samples under examination were analyzed by high-performance thin layer chromatography (HPTLC), in order to identify the polar compounds present. The HPTLC analyses made it possible to obtain a fingerprint of the extracts under examination.

**Figure 1:**
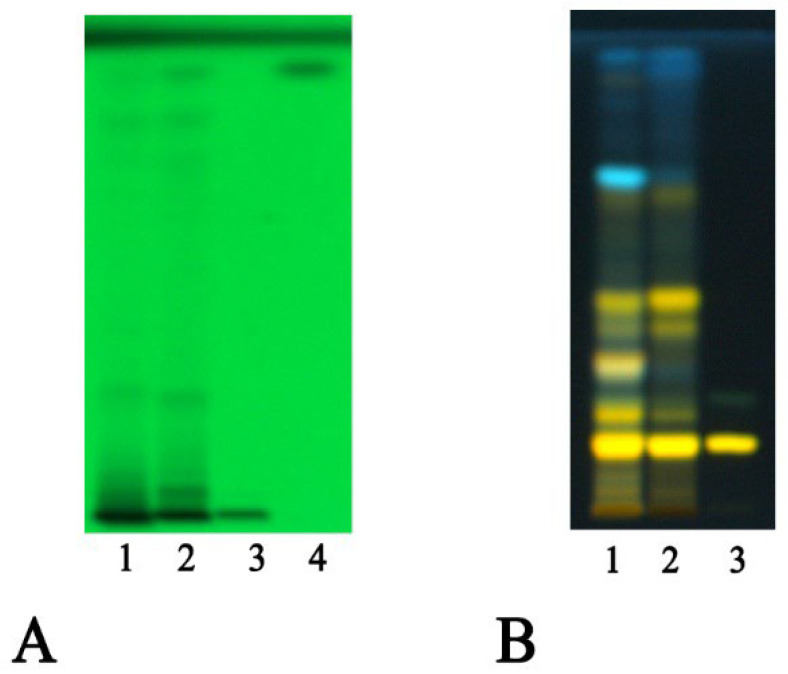
HPTLC analysis of leaf and fruit extracts of *P. spina christi* under examination. (**A**). Traces: 1, leaves; 2, fruits; 3, rutin; 4, gallic acid. Mobile phase: ethyl acetate/dichloromethane/acetic acid/formic acid/water (40:12.5:0.5:0.5;0.5 *v*/*v*/*v*/*v*/*v*). Display: 254 nm. (**B**). Traces: 1, leaves; 2, fruits; 3, rutin. Mobile phase: ethyl acetate/dichloromethane/acetic acid/formic acid/water (100:25:10:10:11 *v*/*v*/*v*/*v*/*v*). Derivatization: NPR; display: 366 nm.

The presence of gallic acid was detected in the ethyl acetate fractions. This phenolic acid appears evident at 254 nm (Figure 1A). Rutin has been shown to be the most abundant flavonoid component (Figure 1B), followed by isoquercetrin and kaempferol 3-*O*-glucoside-7-*O*-rhamnoside. The glycosidic flavonoid rutin was detected in the AcOEt fraction of both extracts and is recognizable by the typical yellow spot after derivatization with Natural Product at 366 nm.

As previous demonstrated phenolic compounds, in particular flavonoids were revealed to be the main components in the leaves, flowers, and fruits [12].

Our results are in agreement also with those of Brantner and Males, who identified rutin as one of the major main flavonoid compounds present in the leaves, flowers and fruits of the plant [16], and those by Zor and coworkers, who described the presence of this phytochemical in the butanol fraction of *P. spina-christi* fruits [26]. Moreover, the presence of gallic acid was also previously reported in the methanolic extract of the stem [27] and the fruits of the plant [28].

### 3.2. Antioxidant Potential

**Figure 2:**
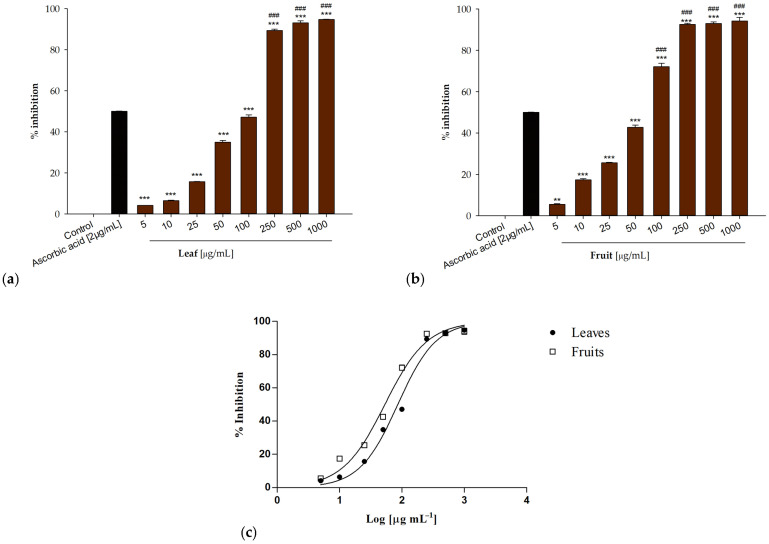
Radical scavenging activity of *P. spina-christi* Mill extracts from (**a**) leaf and (**b**) fruit. (**c**) Non-linear regressions curves. Data are expressed as mean ± S.E.M. (n = 3). Ascorbic acid (2 µg/mL) was used as positive control. Significant difference versus control: *** *p* < 0.001; ** *p* < 0.01; Significant difference versus positive control: ^###^ *p* < 0.001.

The in vitro antioxidant potential of *P. spina christi* extracts was assessed using the DPPH radical scavenging assay and the β-carotene bleaching test. The radical scavenging activity of the samples examined was evaluated using the DPPH test, a colorimetric assay. The DPPH (2,2-diphenyl-1-picrylhydrazyl) radical has a characteristic absorption at 517 nm which decreases significantly when this molecule comes into contact with radical scavenger substances which transform the radical into its reduced form. Both extracts showed a concentration-dependent radical scavenging activity. At the highest tested concentration (1000 µg/mL), both samples induced a 94% inhibitory activity (Figure 2a,b). At 100 µg/mL inhibitory percentages equal to 47.15 ± 1.14% and 72.14 ± 1.55% were detected for leaf and fruit extracts, respectively.

By testing different concentrations, it was possible to calculate the IC_50_ values shown in Table 1.

The best antiradical activity was observed for the fruit extract with an IC_50_ value of 53.41 ± 1.24 µg/mL. The extract obtained from the leaves showed the lowest activity (IC_50_ = 86.06 ± 1.92 µg/mL) (Figure 2c).

The study of Zengin et al. [12] analyzed different solvent extracts of *P. spina-christi* and methanol extract was found to be the most effective antioxidant as evidenced by its DPPH and ABTS scavenging activities. We cannot compare the activity because the method used to evaluate the DPPH radical scavenging activity was different. In particular, our results are in contrast to this study, in which methanol extract from the leaves showed greater antioxidant activity than methanol extract from fruits, using different methods. The study of Takım and Isık [11] revealed that the aqueous fruit extract displayed a high rate of antiradical activity. In this study a different method of extraction was performed, and the analysis was conducted in diabetic rats.

The radical scavenging activity of the investigated samples is higher than that reported for the water and ethanolic extracts from *P. spina-christi* fruits and leaves collected in Turkey, as the highest inhibition percentages for these samples was about 87.4% at the tested concentration of 800 µg/mL [9].

**Figure 3:**
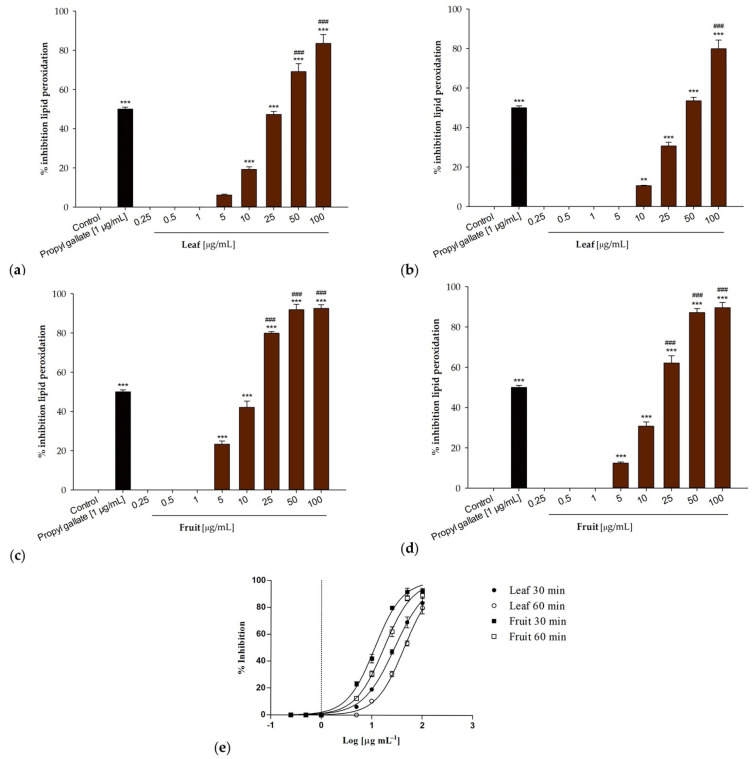
Concentration-dependent inhibition of lipid peroxidation induced by *P. spina-christi* Mill extracts: (**a**) leaf extract after 30 min of incubation, (**b**) leaf extract after 60 min of incubation, (**c**) fruit extract after 30 min of incubation, (**d**) fruit extract after 60 min of incubation, (**e**) Non-linear regressions curves. Data are expressed as mean ± S.E.M. (n = 3). Propyl gallate (1 µg/mL) was used as positive control. Significant difference versus control: *** *p* < 0.001; ** *p* < 0.01; Significant difference versus positive control: ^###^ *p* < 0.001.

The antioxidant activity of the samples under examination was also evaluated through a second in vitro test, the β-carotene bleaching test. This technique consists of measuring the discoloration (bleaching) of β-carotene due to oxidation caused by the degradation products of linoleic acid. All samples were tested at different concentrations (from 0.25 to 100 µg/mL). In line with the previous results, an excellent percentage of inhibition was found in the extract obtained from the fruits with values of 92.48 ± 1.94% after 30 min and 89.57 ± 2.65% after 60 min of incubation at a concentration of 100 µg/mL (Figure 3). This extract has a better inhibitory capacity on lipid peroxidation, with an IC_50_ value of 11.45 ± 0.60 µg/mL after 30 min and 17.38 ± 1.21 µg/mL after 60 min of incubation (Table 1). However, the extract obtained from the leaves showed good activity, with IC_50_ values equal to 28.21 ± 1.66 µg/mL after 30 min and 43.61 ± 2.51 µg/mL after 60 min of incubation (Figure 3).

Phytochemical compounds from plants such as phenols, in particular flavonoids, are the most common constituents responsible for the antioxidant activity. This property is due to the presence in the B-ring of flavonoids structure of hydroxyl groups that can donate hydrogen atoms during free radical reaction. The mechanism of action of phenolics could be different for this reason it is better to use different methods to evaluate the antioxidant activity [29].

### 3.3. Pancreatic Lipase Inhibitory Activity

**Figure 4:**
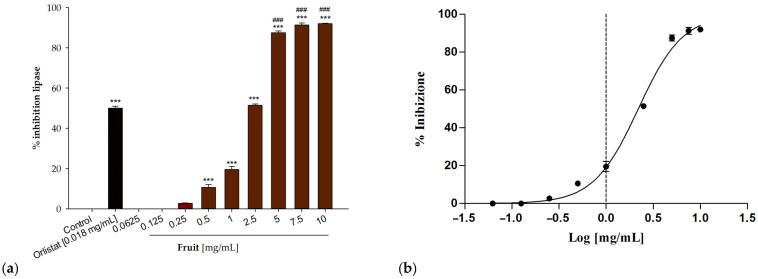
(**a**) Concentration-dependent lipase inhibitory activity of *P. spina-christi* Mill fruit extract. (**b**) Non-linear regressions analysis. Data are expressed as mean ± S.E.M. (n = 3). Orlistat (18 µg/mL) was used as positive control. Significant difference versus control: *** *p* < 0.001; Significant difference versus positive control: ^###^ *p* < 0.001.

There is no longer any doubt about the strong correlation between the alteration of lipids, the complications related to obesity, and the increased incidence of coronary and cerebrovascular diseases. As a result, it is evident that being overweight has a significant impact on the state of health and quality of life of the individual. In this contest, we wanted to evaluate the effect of the samples under examination on pancreatic lipase, the enzyme responsible for the chemical digestion of lipids in the gastro-intestinal tract. For this purpose, the inhibitory activity against this enzyme has been evaluated by monitoring the hydrolysis of p-NPC. As shown in Table 2, excellent activity was observed for the extract obtained from the fruits, which at the highest tested concentrations, 10 and 7.5 mg/mL, induced over 90% inhibition (Figure 4). An IC_50_ value of 2.19 ± 0.02 mg/mL was calculated for this sample (Table 2). In contrast, the extract obtained from the leaves showed no biological activity.

By analyzing the results of radical scavenging, lipid peroxidation, and inhibition of pancreatic lipase experiments, we found a strong correlation between antioxidant properties and pancreatic inhibitory activity. In particular, the extract obtained from the fruits, which showed the best antiradical activity and the best inhibition of lipid peroxidation, also exhibited the most significant inhibition of pancreatic lipase activity. It can therefore be assumed that the fruit extract contains an optimal concentration of specific compounds, in particular flavonoids. Although, as we have previously demonstrated [30] rutin has a considerable effect on lipid absorption and metabolism, we can conclude that other active constituents significantly contribute to the inhibitory activity against lipase.

### 3.4. Molecular Docking

In order to verify whether the biological activity of the extract could be attributed to a direct interaction of its main components with HPL, molecular docking studies were performed on a crystallographic structure of the enzyme retrieved from the PDB (code 1LPB). In this structure, the target protein is complexed with two molecules of MUP, a previously identified C11 alkyl phosphonate inhibitor [20]. In the protein structure, it is possible to distinguish three domains: a C-terminal domain, which includes a colipase binding site, a lid loop that regulates substrate entry, and an N-terminal domain containing the active site characterized by the presence of a catalytic triad (Ser152, Asp176 and Hys263) actively involved in lipid hydrolysis [31]. The mode of interactions of each compound with the enzyme active site was investigated by a protein-based approach, using a protocol already adopted for the study of other polyphenols [32,33].

**Figure 5:**
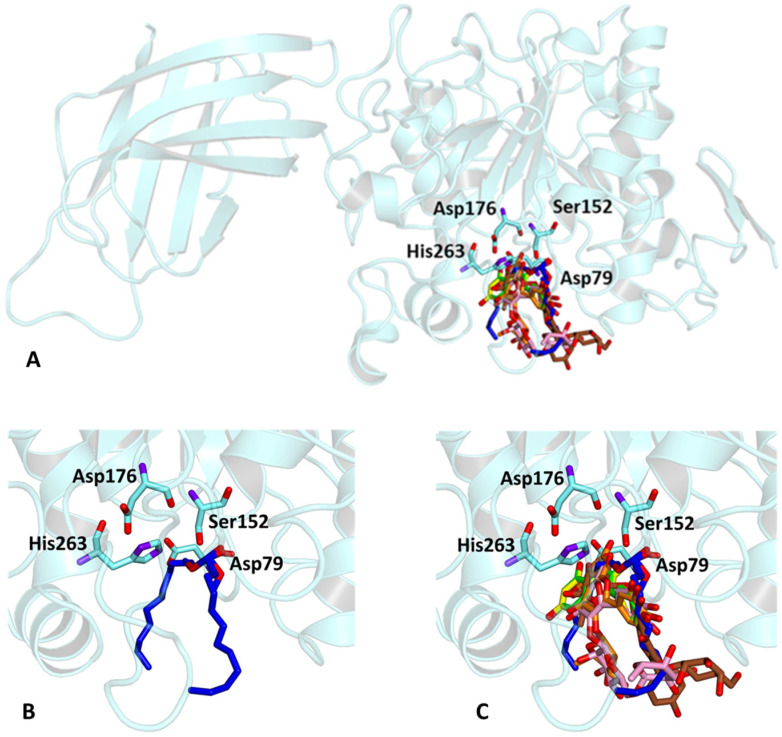
(**A**) Crystallographic structure of pancreatic lipase corresponding to the PDB entries 1LPB. Protein backbone is represented in background as ribbons and key amino acid residues of the catalytic site are highlighted in cyan. (**B**) Binding pose of the crystallographic ligand MUP and (**C**) Superimposed binding modes of the ligands: rutin (pink); quercitrin (gold); kaempferol 3-*O*-glucoside-7-*O*-rhamnoside (brown); quercetin (yellow); kaempferol (green).

Accordingly, the five studied compounds were docked into 1LPB and all of them accommodate the same region of the crystallographic ligand corresponding to the catalytic site of the enzyme (Figure 5).

The pre-docked ligand-protein complexes were subjected to energy minimization, and, as a result of our simulations, all compounds were found to be capable of establishing several hydrogen bonds as well as hydrophobic and π-stacking interactions with residues within the active site of the enzyme, thus contributing to the stabilization of the complexes. These findings are consistent with the estimated binding energy values, which gave very favorable results, ranging from −8.2 to −9.8 kcal/mol (Table 3).

In particular, all ligands bind the catalytic pocket with a similar orientation, such that the chromenone group protrudes towards the inner region of the binding site. In the case of kaempferol 3-*O*-glucoside-7-*O*-rhamnoside, the presence of the rhamnoside moiety at position 7 prevents the chromene group from reaching the same depth as the other compounds in the catalytic pocket. Anyway, this does not hinder the molecule from establishing two hydrogen bonds with Ser152 and His263, both residues belonging to the catalytic trial.

**Figure 6:**
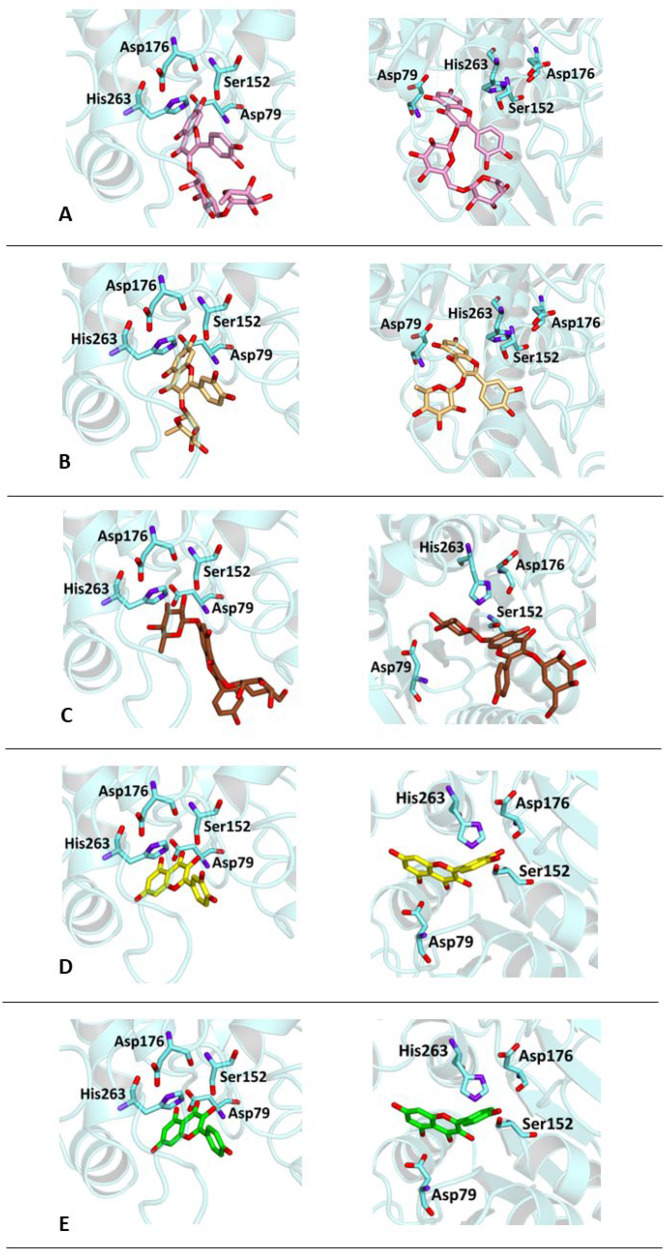
Best docking poses of ligands (**left**) same view of the crystallographic ligand MUP (see Panel B, Figure 5) and (**right**) best view of the same pose: (**A**) Rutin (pink); (**B**) quercitrin (gold); (**C**) kaempferol-3-*O*-glucoside-7-*O*-rhamnoside (brown); (**D**) quercetin (yellow); (**E**) kaempferol (green).

With the exception of this compound, all the other ligands establish hydrogen bonding with His151, whereas all of them interact through hydrophobic interactions with Phe77 and Phe215. Furthermore, both the aglycones quercetin and kaempferol form hydrogen bonds with His151, Ser152, and Arg256. For these compounds, the formation of additional hydrophobic interactions with Phe77, Phe215, Ala260, and Leu264 together with π-stacking interactions with His263 contribute to the complex stabilization. A detailed analysis of the docking results for the best pose of the molecules is reported in Table 3, while the best docking conformations are depicted in Figure 6.

Overall, these findings suggest that all compounds are able to fit into the active site of the target protein, sharing a similar orientation. Furthermore, the binding energy values calculated for quercetin and kaempferol (−9.8 and −9.6 kcal/mol, respectively) are higher than those obtained for glycosides, which would seem to confirm that the biological activity is most likely due to the aglycone portion of the phytochemicals. It is known, in fact, that the majority of flavonoid glycosides are hydrolyzed at the intestinal or hepatic level; therefore, the corresponding free aglycones entering the systemic bloodstream at higher concentrations are able to reach the target site and exert biological activity [34,35,36]. All these data are consistent with the results obtained by biological experiments.

## 4. Conclusions

This study demonstrated that *P. spina-christi* extracts contain important active constituents responsible for their biological activity. We confirmed the antioxidant activity as previously demonstrated using different methods. Furthermore, we demonstrated for the first time the potent lipase inhibitor activity of the extract from fruits.

These biological properties can be supported by the results of molecular docking experiments conducted on the crystallographic structures of lipase. Indeed, our experiments confirm that the most abundant phytocompounds of extracts are suitable ligands of the active site of the protein. The calculated favorable binding energy values support the formation of stable complexes especially for aglycones quercetin and kaempferol. Overall, the results obtained in this study highlight the potentiality of *P. spina-christi* fruit extract and their main constituents as promising candidates for the development of new nutraceuticals, useful as adjuvants for the prevention and/or treatment of metabolic disorders. In contrast, the extract obtained from the leaves showed no activity. Nonetheless, more in vitro, in vivo, and clinical studies need to be carried out, as well as determination of the bioavailability and toxicity profile of the species with particular reference to the fruits.

## Figures and Tables

**Table 1:** IC_50_ values of antioxidant activity of *P. spina-christi* extracts.

Sample	IC_50_ (μg/mL)		
	DPPH	β-Carotene	
		30 min	60 min
Leaves	86.06 ± 1.92 ^b^	28.21 ± 1.66 ^b^	43.61 ± 2.51 ^c^
Fruits	53.41 ± 1.24 ^c^	11.45 ± 0.60 ^c^	17.38 ± 1.21 ^d^
Ascorbic acid *	2.00 ± 0.01 ^a^	-	-
Propyl gallate *	-	1.00 ± 0.02 ^a^	1.00 ± 0.02 ^a^

Data were expressed as mean ± S. E. M. (n = 3). Different letters along column (DPPH) or between columns (β-carotene bleaching test) indicate statistically significant differences at *p* < 0.05 (Bonferroni post-hoc test). * Positive controls.

**Table 2:** IC_50_ Values of Inhibition of pancreatic lipase of the extracts under examination.

Sample	IC_50_ (mg/mL)
Leaf	n.a.
Fruit	2.19 ± 0.02
Orlistat *	0.018 ± 0.001

Data were expressed as mean ± S. E. M. (n = 3). * Positive control.

**Table 3:** Complexes binding energy values and key protein residues of HPL (PDB code 1LPB) interacting with the ligands.

Ligand	Structure	BE *	Interactions					
			HB ***				*^v^HPI	π Stacking
			Res **	Distance Å		Donar Angle °		
				H-A	D-A			
**Rutin**	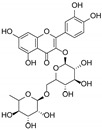	−8.3	Asp79 Tyr114 His151	2.20 2.57 2.65	3.04 3.32 3.43	144.05 134.04 136.86	Phe77 Ile209 Leu213 Phe215	Phe215
**Quercitrin**	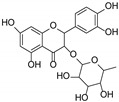	−8.2	Asp79 Tyr114 His151	2.63 3.71 2.54	3.04 4.06 3.37	107.27 105.56 142.30	Phe77 Phe215 Ala259	Phe215
**Kaempferol** **3-** ***O*** **-glucoside-** **7-** ***O*** **-rhamnoside**	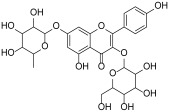	−8.2	Tyr114 Thr115 Ser152 His263	3.02 2.14 2.72 2.44	3.98 2.92 3.66 3.25	169.28 136.13 164.32 139.61	Phe77 Tyr114 Phe215 Ala260	Tyr114
**Quercetin**	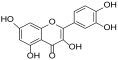	−9.8	His151 Ser152 Arg256 His263	3.21 2.27 2.49 2.88	3.74 3.06 3.05 3.77	114.82 138.20 115.70 153.16	Phe77 Phe215 Ala 260 Leu264	Phe215 His263
**Keampferol**	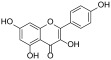	−9.6	Asp79 His151 Ser152 Arg256	2.48 3.10 2.37 2.40	3.29 3.65 3.15 2.99	140.19 117.11 137.17 117.97	Phe77 Phe215 Ala 260 Leu264	His263

* BE = binding energy (kcal/mol); ** Res= residues; *** HB= Hydrogen Bonds; *^v^HPI = Hydrophobic Interactions Res.

## Data Availability

The data presented in this study are available in the article.
